# Symptomatic bradycardia due to nicotine intoxication

**DOI:** 10.5935/0103-507X.20180018

**Published:** 2018

**Authors:** Jin Hui Paik, Soo Kang, Areum Durey, Ji Hye Kim, Ah Jin Kim

**Affiliations:** 1 Department of Emergency Medicine, Inha University School of Medicine - Incheon, Republic of Korea.

**Keywords:** Electronic cigarettes/adverse effects, Nicotine/poisoning, Atropine, Bradycardia

## Abstract

Nicotine is a dangerous substance extracted from tobacco leaves. When nicotine is absorbed in excessive amounts, it can lead to respiratory failure and cardiac arrest. The commercialization of electronic cigarettes (e-cigarettes) has allowed users to directly handle e-cigarette liquid. Consequently, the risk of liquid nicotine exposure has increased. We describe our experience of managing the case of a patient who orally ingested a high concentration of liquid nicotine from e-cigarette liquid. The patient presented with bradycardia and hypotension, which are symptoms of parasympathetic stimulation, together with impaired consciousness. He recovered following treatment with atropine and a vasopressor.

## INTRODUCTION

Nicotine is a dangerous substance extracted from tobacco leaves, and when it is ingested in excessive amounts, it can lead to respiratory failure and cardiac arrest. Nicotine poisoning symptoms result from exposure to normal cigarettes or tobacco leaves. Although there have been reports overseas of nicotine poisoning symptoms resulting from liquid nicotine exposure, clinical reports of liquid nicotine poisoning among Korean adults are lacking.

Since e-cigarettes have been commercialized in Korea, users have direct access to e-cigarette liquid from e-cigarette stores, which has led to an increased risk of exposure to liquid nicotine. E-cigarette liquid, which contains a mixture of nicotine and scents, is more expensive than pure liquid nicotine; therefore, many individuals buy liquid nicotine and add the scents themselves.

Clinically, liquid nicotine poisoning presents similarly to nicotine poisoning caused via other routes. Severe nicotine poisoning has a characteristic biphasic response, with initial excitatory symptoms, such as salivation, nausea, increased bronchial secretions, tachycardia, hypertension, anxiety, muscle spasms, and seizures, followed by symptoms of paradoxical inhibition, including dyspnea, bradycardia, hypotension, lethargy, and paralysis. In rare cases, rhabdomyolysis can develop as a complication.^(1)^

According to the National Poison Data System (NPDS), 169 cases of intentional liquid nicotine poisoning were reported in 2015.^(2)^ There have been no clinical reports of intentional liquid nicotine poisoning by oral ingestion in Korea thus far. We believe that this is the first reported case of intentional liquid nicotine poisoning by oral ingestion in a Korean adult.

## CASE REPORT

A 53-year-old male with no known medical illness was discovered in a state of impaired consciousness, and the emergency medical service was called immediately. At the time, a commercial liquid nicotine bottle was found together with a cup filled with liquid suspected to be nicotine. The patient showed symptoms of impaired consciousness, diarrhea, and vomiting.

The emergency medical service providers arrived approximately 50 minutes after ingestion and found that the patient was conscious enough to respond when called. His vital signs included a blood pressure of 120/60mmHg, a pulse rate of 71 beats/minute, a respiratory rate of 18 breaths/minute, a body temperature of 36.5ºC, and an oxygen saturation of 100%.

It took 15 minutes to transport the patient to the emergency medical center at the hospital. Upon arrival, his vital signs deteriorated to the following: a blood pressure of 96/62mmHg, a pulse rate of 56 beats/min, and a respiratory rate of 22 breaths/minute. Oxygen saturation was maintained at 100%, while his body temperature was 36.0ºC. At this point, although the patient responded to questions, he could not open his eyes properly, and he was sweating profusely. He complained of dyspnea, nausea, and severe generalized weakness.

On physical examination, his pupil sizes were normal and his pupillary light reflexes were intact. His lung sounds were clear bilaterally, and his bowel sounds were slightly increased. Electrocardiography revealed sinus bradycardia, with a QTc of 436ms. An initial arterial blood gas analysis showed a pH of 7.65, a PaCO_2_ of 12.2mmHg, a PaO_2_ of 117.0mmHg, a bicarbonate of 13.5mmol/L, and a base excess of -4mmol/L. General blood test results showed a white blood cell count of 11,970/mm^3^, a hemoglobin of 15.5g/dL, a hematocrit of 44.8%, and a platelet count of 320,000/mm^3^. Electrolyte testing revealed a serum sodium level of 141mEq/L, a potassium level of 3.8mEq/L, and a chloride level of 102mEq/L. The anion gap, lactic acid, and ketone body levels were 13.6mEq/L, 6.1mmol/L, and 176µmol/L, respectively. General blood chemistry test results showed that the levels of blood urea nitrogen, creatinine, aspartate transaminase, alanine transaminase, creatinine kinase, creatine kinase MB fraction (CK-MB), and troponin-I were within normal limits (Table 1). A blood cotinine concentration of 1,296ng/mL was measured at the time of arrival at the emergency medical center (Siemens Immulite 2000 XP-I, Siemens Nicotine metabolite).

**Table 1:** Hemodynamic and laboratory variables over time in a case of acute nicotine poisoning

Time	Baseline*	6 hours	12 hours	24 hours	40 hours
Systolic blood pressure (mmHg)	96	116	125	127	130
Diastolic blood pressure (mmHg)	62	72	77	77	86
Heart rate (beats/min)	56	79	64	54	60
Respiratory rate (breaths/min)	22	18	18	31	20
Body temperature (ºC)	36	37.2	36.8	36.4	36.7
Dopamine dose (µ/kg/min)	0	10	7	0	0
Central venous pressure (cmH_2_O)		8			
Cumulative infusion volume (mL, for 24 hours)	0	1430	2400	3360	
pH	7.65	7.38	7.41	7.4	
PaCO_2_(mmHg)	12.2	30.4	36	37	
PaO_2_(mmHg)	117	105	89	93	
HCO_3_^-^(mmol/L)	13.5	17.4	22.8	22.9	
Base excess (mmol/L)	-4	-6.1	-1.4	-1.6	
O_2_ saturation (%)	99.1	97.4	97	97	
Na^+^(mEq/L)	140	141	141		
K^+^(mEq/L)	3.4	4.1	3.9		
Cl^-^(mEq/L)	113	115	106		
Lactic acid (mEq/L)	6.1	5.2			
Hemoglobin (g/dL)	15.5		13.9		
Hematocrit (%)	44.8		42		
WBC (/mm^3^)	11,970		13,190		
Platelet (×10^3^/mm^3^)	320		259		
BUN (mg/dL)	14.1		9.7	9.9	
Creatinine (mg/dL)	0.8		0.58	0.76	
AST (IU/L)	17		18	23	
ALT (IU/L)	18		19	19	
CK (IU/L)	45		127	126	
CK-MB (ng/mL)	0.8				
Ketone body (µmol/L)	176				

pH - potential of hydrogen; PaCO_2_ - partial pressure carbon dioxide; PaO_2_ - partial pressure oxygen; HCO_3_- - bicarbonate; O_2_ - oxygen; Na^+^ - sodium; K^+^ - potassium; Cl^-^ - chloride; WBC - white blood cell; BUN - blood urea nitrogen; AST - aspartate transaminase; ALT - alanine transaminase; CK - creatinine kinase; CK-MB - creatinine kinase MB.

* Arrival at the emergency department.

Liquid nicotine poisoning was suspected, and the patient was administered a normal saline infusion for the hypotension and lactic acidosis and a single dose of atropine (0.5mg) for symptoms of parasympathetic stimulation, namely, bradycardia, sweating, tachypnea, and salivation. The patient was given 50g of activated charcoal; thereafter, he was able to open his eyes, and his systemic weakness gradually improved. After 10 minutes, his symptoms of dyspnea, sweating, and salivation also showed improvement. Therefore, more atropine was not added. At this time, his blood pressure was 100/60mmHg, his pulse rate was 78 beats/minute, his respiration rate was 24 breaths/minute, and his oxygen saturation was 100%. A central venous catheter was inserted to measure his central venous pressure (8cmH_2_O). The volume of normal saline required for 6 hours of resuscitation was 1,960mL.

**Figure 1:**
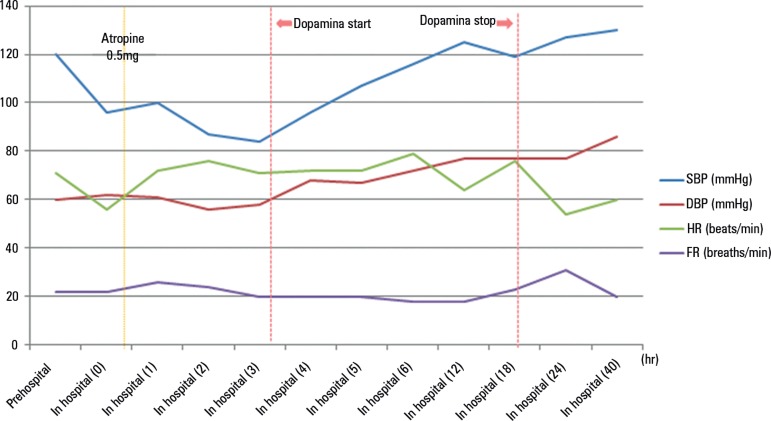
The progression of systolic blood pressure, diastolic blood pressure, heart rate, and respiratory rate from the pre-hospitalization phase to hospital discharge in a case of acute nicotine poisoning. SBP - systolic blood pressure; DBP - diastolic blood pressure; HR - heart rate; RR - respiratory rate.

The patient was given dopamine and admitted to the intensive care unit for observation. While hospitalized, the patient stated that he had acquired liquid nicotine for e-cigarettes (trade name: Pure Nicotine) from an acquaintance and that he had ingested 3mL of this liquid with the intention of committing suicide. The patient's blood pressure normalized within 18 hours of admission, and he was discharged after 3 days (Figure 1).

## DISCUSSION

We report a case of acute nicotine poisoning in a patient who orally ingested a high concentration of liquid nicotine used in e-cigarettes. There have been several studies on nicotine poisoning including cases of unintentional exposure involving workers in the cigarette industry who developed green tobacco sickness and cases of children who ingested cigarettes.^(3,4)^ Cases of poisoning due to intentional exposure have included people who ingested nicotine solution extracted from cigarettes and who simultaneously used multiple nicotine patches.^(5)^

Cases of poisoning by liquid nicotine have been reported since the commercialization of e-cigarettes in 2004, and since the NPDS began recording data for e-cigarettes in 2010, there has been an increase in the number of cases every year.^(2)^

In 2010, Solarino et al. measured nicotine and cotinine levels in a patient who died after ingesting liquid nicotine and found a concentration of 2,200ng/mL.^(6)^ Chen et al. described a patient who had blood nicotine and cotinine levels of over 1,000ng/mL with prolongation of the QTc on electrocardiography upon admission to the emergency room. The patient presented with myoclonic jerking and cardiovascular collapse before dying 3 days later.^(7)^ Sommerfeld et al. reported a case of intentional ingestion and a case of intravascular injection. The patient who underwent treatment after ingesting 372mg of liquid nicotine showed initial symptoms of confusion and vomiting, followed by delayed symptoms of hypotension, upper eyelid weakness, and pale skin. However, he recovered and was discharged after 40 hours of treatment.^(8)^

In the present case, the patient exhibited tachycardia, vomiting, diarrhea, and sweating without hypotension before admission to the emergency room. However, 1 hour after ingestion, he developed bradycardia, hypotension, and severe weakness. While the patient showed no impairments in autonomous breathing or oxygen saturation, he had dyspnea and difficulty opening his eyes due to weakness. The patient reported an improvement in the subjective dyspnea after being given atropine, a change for which three possible explanations can be hypothesized. The dyspnea may have been caused by increased bronchial secretions, which was alleviated by atropine. Alternatively, the dyspnea may have resulted from impairment in respiratory muscle function due to the action of nicotine at the neuromuscular junction, an effect that may have faded over time.

Additionally, the dyspnea may have been caused by metabolic acidosis due to lactic acidosis. The patient's blood gas analysis showed an anion gap metabolic acidosis with respiratory alkalosis. Additionally, his lactic acid levels were increased. We were not able to ascertain if the lactic acidosis was due to shock or the direct effects of nicotine.

Schneider et al. estimated that the total dose of nicotine that could result in the death of an adult was 40 - 60mg; however, they also provided two reasons why it was possible to survive after ingesting a greater dose. First, because nicotine stimulates the vomiting reflex, the actual amount of nicotine that is absorbed is smaller; second, there are individual differences in nicotine metabolism and, particularly for oral ingestion, the first-pass effect decreases the bioavailability of nicotine by 30 - 40%.^(9)^

In the present case, the patient ingested liquid nicotine from a bottle labeled "Pure Nicotine," with no precise concentration indicated. Since there was no liquid remaining in the bottle, it was not possible to accurately measure the concentration; however, according to the study by Kim et al., the nicotine concentration of commercially available liquid nicotine in Korea under the trade name of "Pure Nicotine" is 150 ± 7.9mg/mL.^(10)^ Assuming that the patient ingested liquid nicotine with a similar concentration, he would have ingested approximately 450mg. Since the patient vomited multiple times immediately after ingestion, the actual dose of nicotine absorbed into his body would have been lower than this.

Nicotine is mostly metabolized in the liver, and the main metabolic product is cotinine.^(11)^ As cotinine remains in the blood for a relatively long time, it is measured when determining the dose of nicotine exposure, and it is also used as a marker to diagnose nicotine poisoning and to evaluate its severity to some degree.^(12)^ However, according to clinical reports on nicotine poisoning, clinical patterns and survival rates do not show a consistent pattern based on cotinine concentration. Sommerfeld et al. reported on a case of acute liquid nicotine poisoning by oral ingestion. The patient had a peak blood cotinine concentration of 4,400ng/mL and survived.^(8)^ However, blood cotinine levels ranging from 900 to 2200ng/mL have been reported in patients who died due to liquid nicotine poisoning.^(6-8)^ We were not able to measure the nicotine concentration in the current patient, but the cotinine concentration measured approximately 1 hour after oral exposure was 1,296ng/mL. This value is not high compared to those reported previously; however, a few studies have shown that this amount of nicotine can be fatal if proper treatment is not provided promptly.^(13)^ Thus, while blood cotinine concentration can be used as a reference for diagnosing and treating acute nicotine poisoning, it is more important to diagnose and treat based on an understanding of the patient's medical history and clinical symptoms.

The management of acute liquid nicotine intoxication consists of the provision of supportive care. The cardiovascular and respiratory symptoms caused by parasympathetic stimulation should be monitored. Normal saline should be administered for hypotension. If hypotension continues, norepinephrine and dopamine should be considered. Atropine should be given for bradycardia or dyspnea due to pulmonary secretion. In the case of dyspnea with pulmonary secretion, 0.5 - 1.0mg of atropine should be given repeatedly in 5 - 10 minute intervals. Seizures are rare; however if they do occur, lorazepam, diazepam, or barbiturate should be administered. Gastric lavage or irrigation should be considered for acute liquid nicotine ingestion because of nicotine-induced vomiting. However, it is better to remove materials that release nicotine.^(14)^ It is also imperative to maintain adequate urine output, as nicotine is excreted into the kidneys. Activated charcoal may be effective in decreasing the concentration of nicotine in the blood because nicotine is enterohepatically circulated.^(15)^

Korean law stipulates that e-cigarette liquids containing a mixture of nicotine and scents must display the nicotine content, in accordance with the Tobacco Business Act and enforcement decree.^(16)^ However, pure nicotine solutions are categorized and managed as poisonous substances, according to the Chemical Control Act and enforcement decree.^(17)^ Due to its classification as chemical substances, when liquid nicotine is sold for use in e-cigarettes, it is not regulated according to the Tobacco Business Act; thus, there is no obligation to display the nicotine content.

## CONCLUSION

Each country has different laws and regulations regarding e-cigarettes; in the United States, the Food and Drug Administration manages cigarette products, including e-cigarettes. In Korea, cigarette-related legislation is currently under the jurisdiction of the Ministry of Strategy and Finance. We believe that this limits profound consideration of the public health risks of cigarettes. Allowing the general public to purchase high-concentrations of liquid nicotine for use in e-cigarettes should be recognized as increasing the risk of acute, life-threatening toxicity. There is a need for additional, multi-faceted regulations that account for the unique risks of e-cigarettes, as they differ from commercial cigarettes.
